# Metadata-Based Privacy Assessment for Mobile mHealth

**DOI:** 10.3390/s26030870

**Published:** 2026-01-28

**Authors:** Alejandro Pérez-Fuente, M. Mercedes Martínez-González, Amador Aparicio, Pablo A. Criado-Lozano

**Affiliations:** 1 Grupo de Investigación en Ingeniería de la Privacidad, Universidad de Valladolid, Paseo de Belén 15, 47011 Valladolid, Spain; mercedes@infor.uva.es (M.M.M.-G.); amador@infor.uva.es (A.A.); pacriado@uemc.es (P.A.C.-L.); 2 Departamento de Enseñanzas Técnicas, Universidad Europea Miguel de Cervantes, Calle Padre Julio Chevalier 2, 47012 Valladolid, Spain

**Keywords:** privacy, metadata, mHealth, data flow, data driven analysis

## Abstract

The widespread adoption of mobile health applications has increased the volume of sensitive personal and physiological data processed through interconnected devices. Ensuring privacy compliance in this context remains a challenge, as existing app stores and privacy labeling systems rely heavily on self-declared information. App-PI is a data-driven ecosystem designed to offer end users with tools they can easily manage and privacy researchers with structured and reliable app metadata. It is designed to automate the collection, analysis, and visualization of privacy-related metadata from mobile applications. Heterogeneous data sources are integrated into a unified repository (App-PIMD), enabling the empirical assessment of privacy risks. The data flow design is critical to ensure that the data used to assess privacy impact is of good quality, as well as the privacy indicators that end users will be offered. It is shown on a popular mHealth application, demonstrating the importance of data flow design in order to be able to obtain, from documents and files created for consumption by an operating system, a set of data and tools ready for consumption by the true recipients of health apps: people.

## 1. Introduction

The rapid proliferation of interconnected mobile and Internet of Things (IoT) devices has transformed the collection and processing of personal data, particularly in the domain of digital health. Mobile health applications (also known as mHealth), wearable sensors, and IoT platforms now operate as integrated ecosystems capable of continuously monitoring physiological, behavioral, and environmental signals. While these technologies enable personalized healthcare and data-driven wellness services, they also amplify privacy and transparency risks by increasing the volume, sensitivity, and contextual diversity of user data being collected and shared [1].

However, the ability of end users to check if the apps they use respect their privacy remains limited by the opacity of data-handling practices across mobile and IoT applications [2,3]. In this context, there is a growing need for systematic, data-driven tools capable of automating the evaluation of privacy-related metadata, able to assist end users to identify potential privacy risks across heterogeneous mobile and IoT environments. That is, to help them to make effective their (GDPR) (General Data Protection Regulation (GDPR), *Regulation (EU) 2016/679 of the European Parliament and of the Council of 27 April 2016 on the protection of natural persons with regard to the processing of personal data and on the free movement of such data, and repealing Directive 95/46/EC (General Data Protection Regulation)*.) right to be informed in a clear understandable manner, and to consent or refuse personal data treatments (right to consent). Existing approaches often target isolated aspects of the problem—such as static analysis of permissions, natural language processing of privacy policies, or crowdsourced transparency reports—but lack integration within a reproducible and interpretable ecosystem [4]. Moreover, it is difficult for mobile privacy researchers to find these non-isolated metadata repositories that can be queried to obtain data for research purposes. This was the situation we faced when we intended to test our own privacy indicators and revised the available datasets and repositories [5,6,7,8]. This issue primarily motivated the work presented in this article, which is also inspired by open infrastructures such as AndroZoo [9].

To address these challenges, App-PI (App Privacy Impact), a modular and extensible ecosystem designed to operationalize automated privacy analysis across mobile and IoT applications was designed. App-PI implements a complete end-to-end data flow—from metadata ingestion and transformation to analysis, verification, and visualization—supported by the App-PIMD repository, which consolidates multi-source privacy metadata. Although the ecosystem is domain-agnostic, it is demonstrated here in the mHealth context, where privacy sensitivity and data heterogeneity are particularly pronounced. This is especially relevant in mHealth environments, which are closely aligned with IoT environments that enable continuous, sensor-driven data collection and connectivity.

The research question that motivates the design of the data flow presented by App-PI is how to make effective the right to be informed of mobile application users in general. In other words, any user, regardless of their profile, should be able to access useful and reliable information about the impact that the applications they use may have on their privacy. Such tools are currently scarce. The generality of the profile refers above all to technical and intellectual capabilities, and the ability to pay for these services. There is no doubt that expert knowledge can provide the best quality information, but it comes at a cost. App-PI proposed a system based on the automatic construction of tools that inform users. Although automation means giving up the best of expert knowledge, it offers the possibility of building affordable tools for any user with adequate information quality to assist them in their self-protection.

The flow and processing of data that supports the construction of indicators and visualization tools for users of health applications is shown. Modular ETL pipelines are used for automated metadata collection, normalization, and integration into the App-PIMD repository. The App-PIMD repository provides researchers and privacy-service developers with accurate, high-quality, up-to-date data that supports empirical analysis, reproducible research, and actionable indicators for users. Two principles guide the design of this data flow. First, to provide reliable, high-quality data so that the information users receive is reliable and useful for protecting their privacy. Secondly, to provide this information in a simple, easy-to-understand way for any user profile, with the same aim. To this end, the construction of indicators and visualization tools is proposed, some of which are presented in this article. The whole process is illustrated through a real-world case study, (*Samsung Health*), chosen because of its popularity between mHealth users.

The remainder of this paper is structured as follows. Section 2 reviews the state of the art and related work in privacy transparency and app analyses. Section 3 details the architecture and data flow of the App-PI ecosystem. Section 4 and Section 6 present the mHealth case study and discuss the empirical findings. Finally, Section 5, Section 7, and Section 8 summarize the results and conclusions and outline future research directions.

## 2. State of the Art and Related Work

### 2.1. Privacy and Digital Health in the Internet of Everything (IoE)

Health and wellness applications represent one of the fastest-growing segments of the digital economy. In 2024, the global revenue of the health app industry reached USD 3.74 billion in global revenue, marking a 9% growth from the previous year (https://www.businessofapps.com/data/health-app-market/, accessed on 7 January 2026). This growing popularity aligns with the wider IoE ecosystem, where wearable and mobile sensors continuously generate sensitive personal and physiological data.

The convergence of medical IoT, data analytics, and cloud ecosystems offers unprecedented opportunities for personalized care but simultaneously multiplies privacy challenges [2,3]. Recent research argues that the success or failure of mobile-health data protection strategies depends not only on technical measures, but on the interplay between contextual system, user, task and other factors, which can result in significant harm to individuals [10].

Since Android 14, Google introduced a new permission model through *HealthConnect* and the *HealthPermissions* group (https://developer.android.com/reference/android/health/connect/HealthPermissions, accessed on 7 January 2026), providing on-device governance of health data exchange. Although this marks progress toward privacy-by-design architectures, it also highlights the lack of independent mechanisms to assess whether health apps use permissions responsibly or consistently with their declarations.

### 2.2. Regulatory and Ethical Frameworks for Health Data Privacy

Beyond technical architectures, privacy assurance in health apps is deeply influenced by evolving regulatory landscapes. The *General Data Protection Regulation* (GDPR) in Europe and the *Health Insurance Portability and Accountability Act* (HIPAA) in the United States define explicit requirements for consent, purpose limitation, and data minimization. Emerging frameworks such as the *EU AI Act* extend these requirements to algorithmic transparency and accountability [11]. Researchers emphasize that ethical oversight in digital health must extend beyond traditional norms, focusing on accountability, fairness, and protection of vulnerable users [12].

The App-PI ecosystem aligns with these principles by operationalizing user’s right to be informed in accordance with GDPR principles in article 5—*Lawfulness, fairness, and transparency*—through data evidence stored in structured repositories.

### 2.3. Automated Privacy Assessment and Transparency Mechanisms

Traditional privacy labeling systems, such as Apple’s *App Privacy Details* and Google Play’s *Data Safety* section, provide self-declared summaries of data practices. However, these mechanisms rely on manual developer input and often lack empirical verification [13,14]. Recent works propose automated transparency pipelines that analyze app binaries, permissions, and network flows to infer actual data usage [15,16,17,18,19]. Approaches such as *PrivacyMeter* [20] and *Polisis* [21] apply natural language processing to privacy policies. However, these developments focus on analyzing app privacy in isolation, without systematically comparing declared and observed behaviors through machine-readable evidence, which can lead to inaccurate assessments of overall privacy.

Literature reviews indicate that the majority of complete privacy assessment approaches available to end users are technically focused, as the isolated approaches discussed in the previous paragraph [22,23]. These approaches overcome the limitations of isolated analyses by combining multiple technical methods (e.g., static and dynamic analysis, network traffic inspection), which makes them efective at identifying privacy risks. However, they are not easily adaptable to address non-technical issues, such as transparency assessments across privacy labels or policies [24,25]. Moreover, these approaches frequently overlook user perspectives and rely on opaque analysis techniques, which has been shown to be an issue when it comes to assessing privacy in mHealth applications [26,27,28]. Those that are more hybrid-focused tend to be organization-centric, such as IoTPrivComp [29] and the NIST Privacy Framework [30]. While these are available frameworks that can be adopted by practitioners, they also do not provide users with tools to assess the impact on their privacy. App-PI complements these efforts with a user-oriented approach.

### 2.4. Metadata Repositories and Privacy Intelligence Infrastructures

Metadata repositories have long supported empirical privacy research [5]. Projects such as AndroZoo [6,9], Exodus Privacy (https://exodus-privacy.eu.org/, accessed on 7 January 2026), and PrivacyGrade [31] enabled large-scale analyses of Android apps by collecting APKs and static privacy signals. However, most of these initiatives focus on code and permissions rather than integrating broader metadata—such as privacy policies or the consistency between declared and actual data practices [5].

Later efforts like AndroVault [7,8] expanded these repositories by adding structured relationships and knowledge graphs among apps. However, these systems are limited in scope, as they do not incorporate modern disclosure elements (e.g., Google Play’s *Data Safety* section) and mostly rely on pre-2017 datasets, which precede key privacy updates such as Health Connect and the current Android permission model [32]. Other repositories [33] capture additional metadata such as code evolution from open-source projects, but this excludes most proprietary apps actually used by end users.

The App-PIMD repository extends these prior efforts by merging heterogeneous sources into a unified, machine-readable schema. As part of the App-PI ecosystem, it acts as a foundational privacy assurance infrastructure, enabling the reproducible and large-scale monitoring of mobile app privacy.

### 2.5. Emerging Approaches: AI-Assisted Privacy Auditing and User-Centric Tools

Recent years have seen the emergence of AI-driven methods for privacy auditing. Building on advances in natural language processing and deep learning, machine learning models are now used, identify potential misuse of sensitive data, and classify apps by privacy risk level [34]. Large language models (LLMs) have also been leveraged to automatically interpret complex privacy policies [35]. On the user side, interactive visual analytics tools—such as *AppCensus* [36]—have demonstrated the value of making permission risks and data flows accessible to non-expert audiences. However, *AppCensus* is now a commercial service and not openly available for research use, which limits its applicability for reproducible studies. Building on this line of work, App-PI integrates an open visualization layer that links transparency metrics with user-oriented dashboards, enabling interpretable and reproducible privacy insights.

### 2.6. Synthesis and Positioning of the App-PI Ecosystem

Across the reviewed literature, several open challenges persist:

1. Incomplete and outdated metadata: existing repositories often lack up-to-date information on privacy declarations, or new permission models.
2. Fragmentation of evidence: repositories and studies often analyze permissions, network behavior, or policies in isolation, lacking integrated pipelines.
3. Limited interpretability for health app users: privacy metrics remain complex and rarely translated into actionable insights for individuals managing sensitive health data.

The App-PI ecosystem addresses these challenges by unifying data ingestion, analysis, and visualization within a coherent architecture. Its modular ETL pipelines, metadata repository (App-PIMD), and assessment tools demonstrate a concrete implementation of computational transparency. By bridging empirical privacy research, and user-centric design, App-PI represents a new stage in the evolution of privacy assurance systems for the mHealth domain.

## 3. mHealth Apps Privacy Assessment in App-PI

In this section, we show mHealth privacy processing, including ETLs, the Privacy Assessment Analysis, or the Visualization Tools [37]. These enhancements allow for a more comprehensive assessment of privacy impact in health applications, as they enable the systematic analysis of app behaviors, data collection practices, and visualization tools that facilitate interpretation and comparison of results.

### 3.1. Ecosystem Components

**Figure 1:**
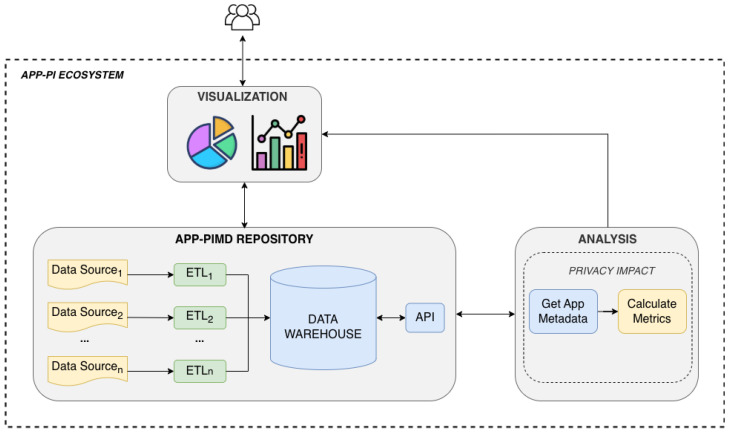
Main components of App-PI ecosystem. Gray boxes correspond to the ecosystem components. Different colors are used for illustrative purposes to distinguish between the various elements.

Several tools collaborate to assess privacy in mobile apps. Figure 1 shows this ecosystem. The central component is a metadata repository, which serves structured metadata to the other components. This is the App-PIMD repository, which hosts metadata related to privacy and security aspects of mobile apps (the repository is freely available to anyone at https://app-pi.infor.uva.es/docs, accessed on 7 January 2026). The *analysis* tools compute privacy indicators and perform transparency assessments. The *visualization* tools present these indicators and results in an accessible, easy-to-understand manner, ensuring that users with diverse technical backgrounds can interpret them. This structured and interoperable approach is particularly valuable when assessing health apps, where data sensitivity and user trust are critical factors.

### 3.2. Component Interactions

The interaction between the App-PIMD repository and the rest of the components follows a structured data flow that starts with the aggregation and structuration of information gathered in the warehouse. Once this data is available, the analysis tools can request it and compute metrics that reflect privacy risk levels. These analyses are stored at the repository so they are accessible to anyone. Finally, the data flow ends when the visualization tools integrate the outcomes of all available metadata and analyses, providing a comprehensive and intuitive representation of privacy indicators that facilitates informed decision-making by end users.

### 3.3. Design of the App-PI Data Flow Supporting mHealth App Privacy Evaluation

Two issues have been of crucial importance for the design of the ecosystem and its data flow: the quality of the data, and the extensibility of the ecosystem. The data entering the ecosystem must come from reliable sources and must not be degraded or corrupted at any stage of its collection and/or processing. Only in this way can the quality of the services provided be guaranteed. The extensibility of the ecosystem refers to its ability to accommodate new components and/or data.

**Figure 2:**
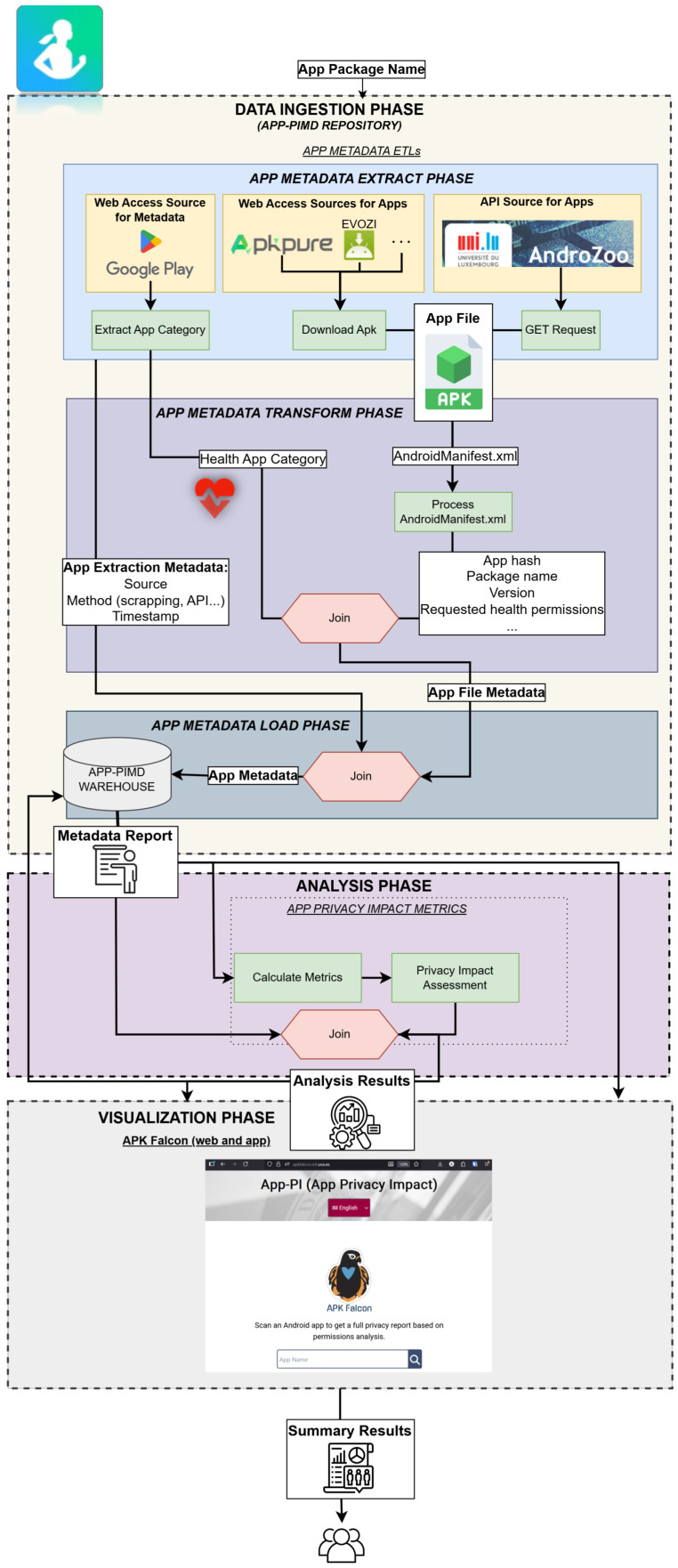
Data flow in App-PI ecosystem.

In Figure 2, the data flow in this ecosystem for the case study of Section 4 is shown. The data flow is structured in several phases, according to the type of tasks performed on data: *Data Ingestion*, *Data Analysis*, and *Data Visualization*.

#### 3.3.1. Data Ingestion Phase

The process begins with the data ingestion phase, in which app metadata are loaded as structured data into the central repository, App-PIMD. This is done by a set of ETL pipelines, which ensure that data values are valid before entering the repository. The ETL processes are used to extract different types of information from multiple data sources, transform them into structured datasets, and store them. The design approach followed ensures the extensibility and adaptability of the ingestion phase to accommodate new categories of data that may emerge in the context of mobile app privacy.

To ensure the repository remains up to date, the ETL pipelines are executed on a monthly schedule. This periodic update ensures that newly released applications and changes in existing ones are incorporated in a timely manner. The process begins by retrieving the most popular apps in each Google Play category, after which the corresponding ETL workflows are launched to process and integrate their metadata into the repository.

Two types of ETLs have been considered. First, to extract the information provided to end users about the personal data collected by the app. These data can be used to assess *transparency* in app’s privacy policies and data safety declarations. Second, to extract app metadata and metadata about the operating system in which apps run. These metadata can be used to obtain indicators that help users to understand potential privacy risks. The following subsections describe the two types of ETL processes implemented in this phase.

##### App Declarations ETLs

1. *Extract Phase.* In this phase, the various app data collection statements are extracted, including privacy policies and, in the case of Android applications, the declarations available in the “Data Safety” section of Google Play (https://support.google.com/googleplay/android-developer/answer/10787469?hl=en, accessed on 7 January 2026).
2. *Transform Phase.* From the raw declaration data, the personal data items collected by the app are extracted and converted into structured, machine-readable formats. This is achieved using pattern matching techniques to identify and classify the types of personal data mentioned in the declarations. To ensure consistency and accuracy, a predefined vocabulary of personal data types is used. This vocabulary is based on the Google Play *Data Safety* section documentation (https://support.google.com/googleplay/android-developer/answer/10787469?hl=en, accessed on 7 January 2026), which provides a reference list of data types that an app may request. Any items not matching the vocabulary are flagged for manual review.
3. *Load Phase.* During this phase, the structured data obtained from the extraction process are ingested into the data warehouse.

##### App Metadata ETLs

1. *Extract Phase.* Apps are downloaded from multiple sources integrated into the warehouse, including APKPure, Evozi, and AndroZoo [6,9]. Source priority is defined to optimize reliability and performance: AndroZoo is queried first due to its inclusion of Google Play apps and efficient API-based access; the remaining sources are consulted according to their relative popularity. Google Play is used to retrieve the app category. Data from AndroZoo are obtained via its API, while other sources are accessed through web scraping, mostly implemented with pattern searches using regular expressions on page links, allowing the process to be easily extended to new sources. Metadata about the extraction process itself (*source*, *method*, *timestamp*) are also stored, allowing each app to be fully traceable. When multiple sources provide the same APK (identical hash), no conflict exists, as all extracted metadata coincide. If different APKs appear to be identical (identical package name and version) but differ in hash, both are preserved to enable further analysis, although we have not observed such cases in practice.
2. *Transform Phase.* Using the APK file and app category as input, the *AndroidManifest.xml* is extracted and analyzed to collect key metadata, such as app hash, package name, version, requested permissions, such as health ones, and other relevant attributes. The requested permissions are compared against a list of known Android permissions to ensure their validity. This list is updated periodically to reflect changes in new versions of the Android OS. If a permission is not found in the list, it is marked as non-Android OS permission, and it is stored separately for further analysis. Any invalid or inconsistent permissions are rejected at this phase.
3. *Load Phase.* All extracted metadata, along with information about the extraction process, are combined and loaded into the warehouse.

#### 3.3.2. Analysis Phase

Metadata stored in the warehouse, previously collected at the *Data Ingestion Phase* (*Metadata Report* in the Figure 2), are analyzed to assess different aspects of app privacy. This phase encompasses two complementary analytical processes. The first performs an *App Declarations Transparency Assessment*, which evaluates the consistency and completeness of the privacy-related statements provided by app developers, such as privacy policies and data safety declarations [38]. We define *consistency* as the alignment between what an application declares in its Privacy Policy, what it reports in the Google Play *Data Safety* section, and the permissions requested in its *AndroidManifest.xml* [38]. Any mismatch among these three sources is considered a *inconsistency*. For example, if the app requests the permission READ_HEART_RATE but does not disclose the collection of heart-rate data in its *Data Safety* section, this is flagged as an inconsistency. The second process focuses on computing *App Privacy Impact Metrics*, derived from the metadata and permissions extracted from the apps. These metrics provide quantitative, easy-to-understand indicators of the potential privacy risks associated with each application [39]. The metrics consider several factors, including the sensitivity of the permissions, the number of permissions requested, and their potential impact on user privacy [39]. The impact of each permission is computed based on cybersecurity expert knowledge [40]. Together, these analyses offer a comprehensive view of both the actual and declared privacy practices of mobile applications, appearing as *Analysis Results* in Figure 2. These results are uploaded to the central warehouse, where they are made available to other components, services and researchers who query the repository.

#### 3.3.3. Visualization Phase

**Figure 3:**
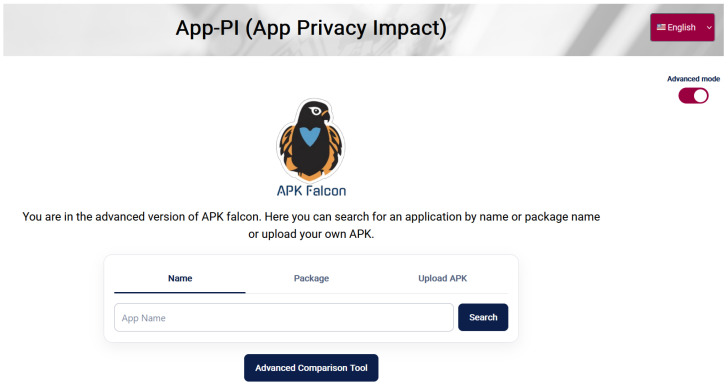
Advanced APK Falcon interface.

This phase presents the results obtained during the *Analysis Phase* (*Analysis Results*) in a clear and easily interpretable manner for end users. For now, one tool has been implemented to support users in understanding the privacy characteristics of mobile applications: *APK Falcon*, as shown in Figure 3 (Available at https://apkfalcon.inf.uva.es/, accessed on 7 January 2026). It is a user service that presents the results of a privacy indicator obtained using the information about permissions requested by an app. The global privacy impact is shown as a numeric value, classified in three categories: Low (green in the figure), Medium (yellow), and High (red). This privacy indicator works only with permission on which users can operate, that is, they can grant or revoke in the Settings of their mobile devices. Therefore, it does not provide an absolute and accurate information about app intrusiveness, but offers information to support end users decision-making: providing users with awareness of potential app impact on their privacy before installing apps on their personal devices. Since its initial development, APK Falcon has evolved into a fully-featured privacy assessment visualization tool, available both as a web site (https://apkfalcon.inf.uva.es/, accessed on 7 January 2026) and a mobile application (currently being uploaded to Google Play Store). It is a multilingual tool (English, Spanish, German, French, and Italian) that allows users to search for applications by name and retrieve detailed privacy information, including the app’s name, version, icon, and a global privacy impact score broken down by permission groups. Users can simulate how granting or revoking permission groups affects the overall impact using interactive charts and explore a table of similar applications within the same category, showing higher and lower privacy impact scores, with the option to select multiple apps for side-by-side comparison.

## 4. Application to a mHealth App: Workflow and Data Flow

This use case illustrates the full execution of the App-PI data flow applied to the *Samsung Health* (*Samsung Health* is mentioned strictly as a case study; the authors have no commercial or institutional relationship with Samsung.) app, available on Google Play (https://play.google.com/store/apps/details?id=com.sec.android.app.shealth, accessed on 7 January 2026). The goal is to demonstrate how the proposed ecosystem supports the privacy evaluation of mobile applications. Version 6.29.2.001 of the application was analyzed (the analyzed version is available in the App-PIMD repository). To select a mobile application we decided to use one of the more widely used criteria in mobile environments to rate the representativeness of apps: number of downloads from Google Play. Unfortunately, we did not find open-source mHealth applications with comparable functionality, adoption, or data collection features that could provide a representative case study.

*Samsung Health* was selected as the representative sample primarily because it is one of the most widely used mHealth applications, with more than one billion installs and approximately 1.55 million user ratings on Google Play (values as of 17 November 2025). This level of adoption makes it a highly representative example of real-world usage patterns and ensures the relevance of the case study. In addition to its widespread use, the app integrates and processes multiple categories of sensitive health data and—according to its own *Data Safety* disclosures—shares such information across different services. This makes it a particularly suitable case for assessing privacy risks. Finally, it constitutes a typical example of proprietary software, which reflects the majority of applications encountered by end users.

The data flow begins with the package name of the application to be loaded (see Figure 2). The package name for the *Samsung Health* application is “com.sec.android.app.shealth” as can be seen at its Google Play URL. This is the only input provided to the App-PI ecosystem in this instance.

### 4.1. Data Ingestion Phase

The App-PIMD repository triggers all the ETLs to start looking for the required information from the different data sources when it receives the package name of the requested application.

#### App Metadata ETLs

The corresponding APK file was obtained from reputable repositories, such as APKPure and AndroZoo, to ensure data quality [6,9]. The *AndroidManifest.xml* file was subsequently extracted and parsed to derive structured metadata, including the application name, version code, and declared permissions. This process identified 75 permissions, which were classified according to Android’s permission model (*Dangerous, Normal, Signature*) [32,41]. Table 1 summarizes these results, indicating that 50 permissions are categorized as *Dangerous*—that is, user-controlled—while the remaining 25 belong to categories not directly manageable by users. Additional metadata, such as timestamp, data source, and retrieval method, are also retained in the warehouse to ensure comprehensive traceability.

All this structured metadata—both the app’s extracted attributes and process metadata, as well as the declarations metadata—was subsequently loaded into the App-PIMD warehouse to support further analysis phases and make it publicly available to others.

### 4.2. Analysis Phase

**Figure 4:**
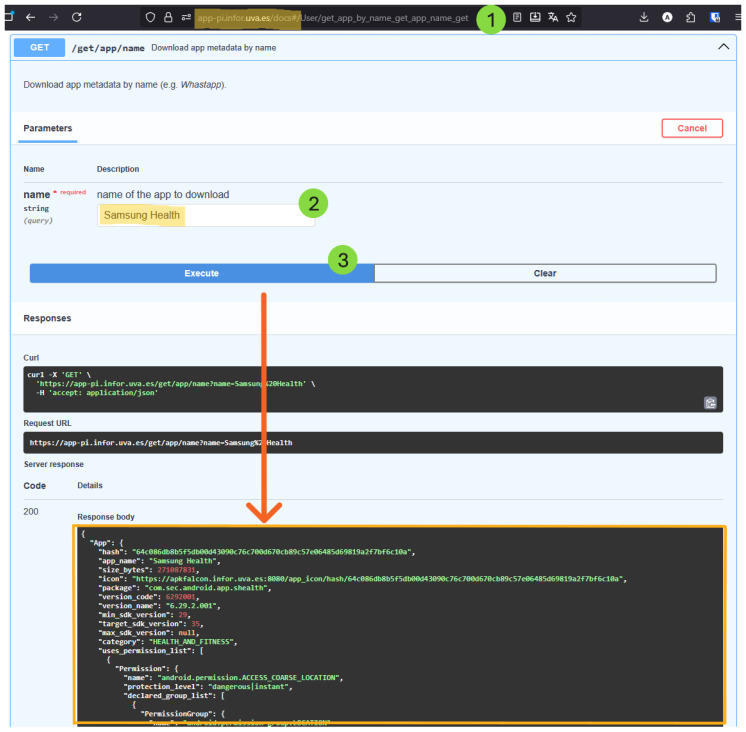
Using App-PIMD to obtain the *Metadata Report*. The numbers correspond to the order of user interactions.

By invoking the App-PIMD endpoint /get/app/name with “Samsung Health”, the *Analysis Phase* retrieves the previously collected *Metadata Report* (see Figure 4). Then, the two complementary analysis phases are executed: the *App Declarations Transparency Assessment* and the *App Privacy Impact Metrics*.

#### App Privacy Impact Metrics

Table 2 presents the estimated privacy risk for each permission group, as calculated by the privacy impact metric, showing both absolute and percentage values [39]. For example, the *Phone* permission group has an impact value of 0.496785, representing 37.75% of the app’s total privacy impact. This value is calculated as the sum of the individual impacts of all *Dangerous* permissions within the *Phone* group that are requested by the app. The overall impact metric is based solely on *Dangerous* permissions, as these are the only permissions that users can control [39]. In this case, the app requests READ_PHONE_NUMBERS, with an impact of 0.488382, and READ_PHONE_STATE, with an impact of 0.008403, which together result in a total impact of 0.496785 for the *Phone* group. The percentages indicate the relative contribution of each permission group to the app’s overall privacy impact. The groups with the greatest relative impact are *Phone* (37.75%) and *Contacts* (30.96%), followed by *Health* permissions (24.27%). Although health permissions are not the largest contributors, they are among the top three, highlighting their significance. The app’s final privacy impact value (0.3219, i.e., 32.19%) is obtained by dividing the sum of impacts of all requested groups (1.3160) by the maximum impact that a hypothetical app requesting every possible *Dangerous* permission would reach (4.088). This indicates that the app is not highly intrusive—an observation that may be somewhat unexpected given the sensitivity of the data handled.

### 4.3. Visualization Phase

Finally, we reach the visualization phase, where all previously extracted, processed, and analyzed data is presented in a clear and accessible manner. In this example, the APK Falcon tool is used to assess the privacy impact of the selected application, *Samsung Health*.

**Figure 5:**
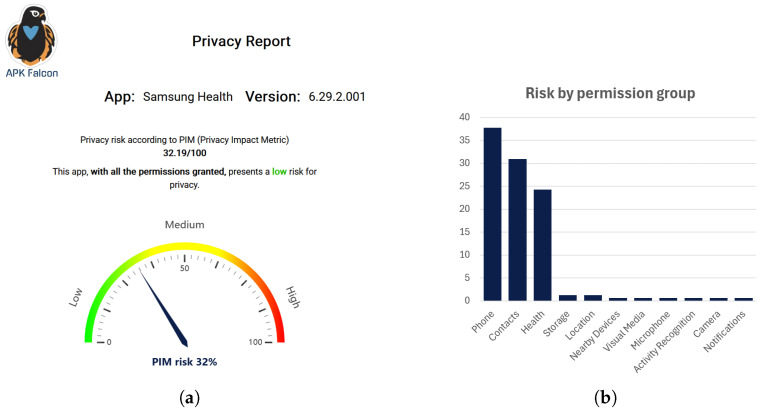
(**a**) Assessment of *Samsung Health*’s privacy impact using APK Falcon. (**b**) Privacy impact broken down by permission group.

Figure 5a presents a screenshot of APK Falcon displaying the extracted *Metadata Report*, including the app name and version, along with the *Analysis Results*; both are publicly available in the App-PIMD repository. The overall privacy impact score is shown both as a numerical value with an explanation and as an intuitive chart using traffic-light–style colors, making it especially accessible to non-expert users. Figure 5b illustrates the contribution of each permission group to the overall privacy metric, allowing users to identify which groups have the greatest impact on their privacy. Furthermore, APK Falcon enables users to simulate changes in the overall impact by allowing or denying specific permission groups, made possible because the privacy impact metric focuses exclusively on *Dangerous* permissions and their groups [39].

## 5. Synthesis of Key Results

This section summaries the key outcomes obtained from both the design of the App-PI ecosystem’s data flow and its application to the *Samsung Health* use case. The aim is to provide a concise overview of the results produced, emphasizing the system’s capacity to ensure data quality, extensibility, and the interpretability of privacy assessment results.

- At ecosystem-level results, the App-PI ecosystem successfully demonstrated the complete execution of its data flow, from ingestion to visualization, relying only on the app package name as input. The modular design enabled the integration of multiple ETL pipelines and analysis tools, ensuring data consistency and traceability across all stages. The quality assurance mechanisms embedded in the ingestion phase contributed to reliable metadata generation, while the visualization tool (APK Falcon) effectively translated analytical results into user-friendly representations. These results confirm the extensibility and operational robustness of the ecosystem.
- The results of the *Samsung Health* use case are detailed below for each phase. The *Data Ingestion Phase* processes extracted and structured both app declarations and metadata from multiple repositories (see Section 4.1). The *Analysis Phase* (Section 4.2) identified a total of 75 declared permissions, of which 50 were classified as *Dangerous*—that is, under direct user control. The computed privacy impact metric yielded a global risk value of 0.3219 (32.19%), indicating a moderate level of intrusiveness. The primary contributors to this score were the *Phone* (37.75%), *Contacts* (30.96%), and *Health* (24.27%) permission groups. These outcomes are visualized through APK Falcon, which presents both the global score and per-group contributions, allowing users to simulate different permission configurations.
- Overall, the results confirm that App-PI provides an automated, traceable, and interpretable workflow for privacy assessment in mHealth applications. It efficiently integrates heterogeneous data sources and produces quantitative and qualitative insights that can support privacy user awareness.

## 6. Discussion

The App-PI ecosystem and its application to the *Samsung Health* case study have demonstrated the feasibility of automated, data-driven privacy assessments for mHealth applications. However, several limitations and considerations must be acknowledged regarding the *Visualization Phase*, and generalizability of the ecosystem.

### 6.1. Visualization of Privacy Indicators

Although our current visualization tools do not yet cover all aspects of the privacy analysis, the modular design of App-PI enables new visual components to be incorporated and integrated seamlessly, strengthening the ecosystem’s value for end users. As introduced in Section 2, existing privacy assessment approaches differ notably in both scope and target audience. Regarding the scope of the analysis, we can distinguish between technical-centric approaches and others that include non-technical elements, i.e., hybrid approaches. Technical-centric solutions based on methods such as static or dynamic analyses overlook the declarative and non-technical elements present in hybrid approaches [16,18,24,25]. Hybrid approaches combine these methods with declared data practices to offer a more comprehensive assessment. These are needed for better evaluations, as explained in Section 2 [17,19,20,21]. However, they still fall short of offering users concrete guidance on how to mitigate risks.

Considering the target audience, we can distinguish between organizational-centric and user-centric approaches. Organizational-centric frameworks provide high-level guidance to companies and public bodies, but they do not focus on offering users interpretable or actionable insights [29,30]. In contrast, user-centric approaches aim to empower end users by providing them with interpretable analyses and actionable recommendations to help them make informed decisions regarding their privacy, such as AppCensus [36]. Compared to these frameworks, App-PI is a hybrid, user-centric approach. AppCensus is the framework that is closest to App-PI, as it is also a hybrid, user-centric approach. However, it is not openly available for research or reproducibility. In contrast, App-PI provides an open, extensible framework that can be used to reproduce analyses and integrate new tools, even from third parties.

### 6.2. Generalizability of Results

The current evaluation successfully demonstrated the ecosystem’s functionality using *Samsung Health* as an initial case study. The App-PI ecosystem is designed to analyze any application, leveraging a robust and scalable architecture that can process diverse apps with varying behaviors, privacy policies, and data-handling practices. Despite the Android version or whether the app utilizes Health Connect, the ecosystem remains effective in assessing privacy impacts. The planned extension of the evaluation to a larger set of mHealth apps will help showcase the ecosystem’s versatility and confirm its value for privacy impact assessment across different application types.

### 6.3. Interpretation of Privacy Metrics

The interpretation of privacy impact metrics also warrants discussion. Although the computed value for *Samsung Health* (32.19%) suggests a moderate level of intrusiveness based on the visualization tool’s predefined thresholds, such categorization should still be interpreted with caution. While these thresholds provide initial guidance for interpreting privacy scores, they are not derived from empirical distributions across large app samples. The current definition of the privacy impact metric yields a normalized value in the interval [0,1], where 1 represents the highest level of intrusiveness according to the metric design [39]. The visualization tool represents this continuous value into *low*, *moderate*, and *high* categories by dividing the interval into three equal segments and scaling the result to a 0–100 range. Future iterations could enhance the reliability of these reference scales by incorporating data-driven thresholds based on statistical analysis of privacy scores across comprehensive app datasets. As an illustrative example of a data-driven approach, future iterations will compute the privacy impact metric for a large and diverse corpus of mobile applications covering multiple categories. Once collected, these values will be analysed to obtain their empirical distribution. Using this distribution, we could derive percentile-based thresholds (e.g., 25th, 50th, and 75th percentiles) to define data-driven boundaries for *low*, *moderate*, and *high* impact. This would allow the visualization tool to ground its categories in observed real-world patterns instead of equal interval splitting, aligning the interpretation of the metric with typical risk levels.

It is also important to note that the current privacy metrics are derived solely from *Dangerous* permissions, which users can grant or revoke manually. While this focus strengthens the ecosystem’s applicability and interpretability—since it aligns with user autonomy—it does not capture all potential privacy risks. Background data transmissions, SDK-level tracking, and undeclared data flows may still occur without requiring user-controlled permissions. Hence, the privacy impact metric should be viewed as a user-centric indicator rather than a comprehensive measure of privacy compliance. Nevertheless, the metric has been validated through statistical analyses, confirming its ability to meaningfully reflect privacy risk levels as they are disclosed by user reviews on similar services [39]. In addition, we are currently conducting user-centred evaluation studies to further validate the metric’s interpretability and practical usefulness for end users.

## 7. Conclusions

The use of the App-PI metadata ecosystem with mHealth applications has been shown. Privacy impact assessment is automated, incorporating the data-driven computation of privacy indicators and end-user interfaces to facilitate end user consumption. The proposed data flow—encompassing ingestion, analysis, and visualization—was demonstrated through the evaluation of *Samsung Health*, confirming the system’s ability to detect inconsistencies between declared and actual data practices and to compute quantifiable privacy impact metrics. The modular ETL pipelines and the App-PIMD repository ensure data quality, traceability, and reproducibility, while the visualization tools (APK Falcon and ApkAudit) translate analytical results into interpretable insights accessible to non-expert users. Overall, the ecosystem consolidates technical, and user-centric dimensions of privacy assurance, contributing for empirical privacy analysis in the mHealth domain.

## 8. Future Work

Future developments include integrating dynamic analysis techniques to complement static verification, Moreover, expanding the transparency visualization layer to merge privacy impact metrics with disclosure-consistency indicators, enhancing user interpretability. These future extensions will reinforce App-PI’s role as an open, data-driven infrastructure for privacy accountability in digital health.

## Figures and Tables

**Table 1:** Number of permissions by protection level.

Permission Type	Number
Dangerous permissions	50
Normal permissions	23
Signature permissions	2
**Total**	75

**Table 2:** Privacy impact of each permission group in the *Samsung Health* app.

Permission Group	Impact	Relative Impact (%)
PHONE	0.496785	37.75%
CONTACTS	0.407408	30.96%
HEALTH	0.319328	24.27%
STORAGE	0.016807	1.28%
LOCATION	0.016807	1.28%
NEARBY_DEVICES	0.016807	1.28%
READ_MEDIA_VISUAL	0.008403	0.64%
MICROPHONE	0.008403	0.64%
ACTIVITY_RECOGNITION	0.008403	0.64%
CAMERA	0.008403	0.64%
NOTIFICATIONS	0.008403	0.64%
**Overall Impact**	**1.3160**	**32.19% **
**Request All Hypothetic App Impact**	**4.0880**	**100%**

## Data Availability

Data analyzed in this article are publicly available in the App-PIMD repository: https://app-pi.infor.uva.es/docs/, accessed on 7 January 2026. Analysis results are available within the article. Other data that support the findings of this study are available from the corresponding author.
